# Structural and electronic properties of SnO_2_ doped with non-metal elements

**DOI:** 10.3762/bjnano.11.116

**Published:** 2020-09-03

**Authors:** Jianyuan Yu, Yingeng Wang, Yan Huang, Xiuwen Wang, Jing Guo, Jingkai Yang, Hongli Zhao

**Affiliations:** 1 College of Materials Science and Engineering, Yanshan University, Qinhuangdao 066004, China; 2 Department of Environmental and Chemical Engineering, Tangshan University, Tangshan, Hebei 063000, China; 3 Graphene Application Technology Tangshan Public Service Platform, Tangshan, Hebei 063000, China; 4 School of Civil Engineering, Tangshan University, Tangshan, Hebei 063000, China; 5 State Key Laboratory of Metastable Materials Science and Technology, China

**Keywords:** density functional theory (DFT), doped SnO_2_, electronic structure, optical properties

## Abstract

Crystal structure and electronic properties of SnO_2_ doped with non-metal elements (F, S, C, B, and N) were studied using first-principles calculations. The theoretical results show that doping of non-metal elements cannot change the structure of SnO_2_ but result in a slight expansion of the lattice volume. The most obvious finding from the analysis is that F-doped SnO_2_ has the lowest defect binding energy. The doping with B and S introduced additional defect energy levels within the forbidden bandgap, which improved the crystal conductivity. The Fermi level shifts up due to the doping with B, F, and S, while the Fermi level of SnO_2_ doped with C or N has crossed the impurity level. The Fermi level of F-doped SnO_2_ is inside the conduction band, and the doped crystal possesses metallicity. The optical properties of SnO_2_ crystals doped with non-metal elements were analyzed and calculated. The SnO_2_ crystal doped with F had the highest reflectivity in the infrared region, and the reflectance of the crystals doped with N, C, S, and B decreased sequentially. Based on this theoretical calculations, F-doped SnO_2_ is found to be the best photoelectric material for preparing low-emissivity coatings.

## Introduction

Thin film solar cells are devices that convert solar energy into electrical energy. Transparent conductive films (TCFs) are a thin film material with both conductive capabilities and high transmittance in the visible light range (300–800 nm) [1–3]. TCFs serve as the front electrode of thin film solar cells. Up to now, the solar energy conversion efficiency is about 23.3% [4], and it is important to increase the photovoltaic power generation efficiency, as well as the performance of the front electrode.

The intrinsic semiconductor SnO_2_ is not conductive due to the absence of free carriers. However, the bandgap of 3.6 eV of SnO_2_ makes it a potentially ideal material for transparent electrode films. It had been proved that the doping of heteroatoms to replace Sn or O can lead to more carriers or holes. Therefore, extensive research works has been done examining different doping elements. Doped tin oxide thin film have been widely used in the fields of thin film solar cell electrodes, electronic display devices, and gas sensors. Also doped SnO_2_ been used for energy-saving low-emissivity glass coatings due to low resistivity, high transmittance of visible light, good thermal stability, acid–base corrosion resistance, high material hardness, and easiness of preparation processes [5]. Non-metal atoms, such as fluorine (F) and nitrogen (N), were proved to be proper elements for doping. After doping with heteroatoms, the preferred orientation, optical properties, and electrical properties of SnO_2_ film are improved.

Analysis of the electrical properties of SnO_2_ films doped with different non-metal elements showed that the resistance of SnO_2_ films doped with N was higher [6–8] than that of SnO_2_ doped with other elements. Nguyen successfully prepared p-type N-doped SnO_2_ films using magnetron sputtering [9]. The results show that the SnO_2_ films were n-type semiconductors, and the concentration of free carriers in the film increased as the temperature for sedimentation increased. Also, p-type semiconductors were successfully prepared from N-doped SnO_2_ films. Through Al/N co-doping, a p-type SnO_2_ semiconductor thin film with excellent electrical properties was prepared. The resistivity, hole concentration and hole mobility were 7.1 × 10^−3^ Ω·cm, 6.24 × 10^19^ cm^−3^ and 14.1 cm^2^·V^−1^·s^−1^, respectively [8]. Doping SnO_2_ with F (substituting O) can effectively increase the carrier concentration and improve the conductivity. Majumder successfully prepared SnO_2_:F thin films using spray pyrolysis with SnF_2_ as the precursor. By adjusting the concentration of the precursor solution, doped SnO_2_ films with different properties were obtained. When the concentration of the precursor solution was adjusted to 0.15 M and the substrate temperature was 773 K, a film with a resistivity of 1.2 × 10^−4^ Ω·cm was obtained [10]. Theoretical calculations, based on first principles, show that the doping of N into the SnO_2_ crystal structure can introduce oxygen vacancies, and thus, increase the charge density of the Sn sites. The replacement of O with N can simultaneously decrease the release of CO_2_.

While there are reports on experiments regarding the doping of SnO_2_ with non-metal elements, the mechanism of the effect of non-metal element doping on the performance of SnO_2_ is not yet clear. In recent years, many researchers used first-principles calculations to scrutinize the doping of SnO_2_ with non-metal elements such as F [11–12] and S [13]. The results show that the optical and electrical properties of SnO_2_ thin films can be changed by doping with different elements. However, the calculation conditions used in these works are uniform, so it is difficult to compare the effects of different doping elements. Also, some results are even inconsistent. We selected several non-metal atoms (N, C, B, F, and S) with an atomic radius similar to that of O to dope SnO_2_. The calculations were carried out with the CASTEP software. In this paper, density functional theory (DFT) is used to analyze electronic structure and optical properties of SnO_2_ doped with non-metal elements.

## Crystal Structure Model and Calculation Method

**Figure 1:**
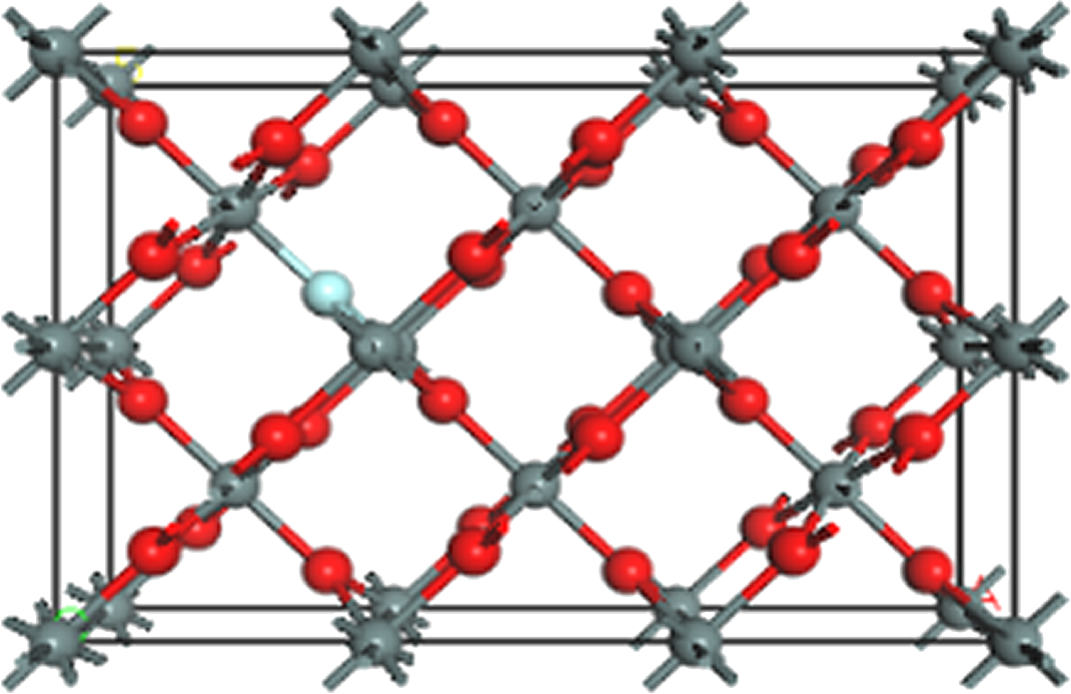
Schematic diagram of the structure of a non-metal atom replacing an O atom in a SnO_2_ unit cell (The red spheres are oxygen atoms, the gray spheres are tin atoms, and the light blue sphere is the non-metal atom).

The SnO_2_ crystal has a tetragonal structure with space group *P*4_2_/*mnm*. There are six O^2−^ ions as nearest neighbors of each Sn^4+^ site, and three Sn^4+^ ions as nearest neighbors of each O^2−^ ion. Hence, the coordination numbers for Sn and O are six and three, respectively. The CASTEP package was used to construct a 3 × 2 × 1 SnO_2_ supercell. To study the effect of non-metal element doping on the structure of SnO_2_, N, C, B, F, or S was used to replace one O atom in the supercell. The model diagram of doped SnO_2_ is shown in Figure 1.

The crystal structure optimization and electronic structure of the doped SnO_2_ cell were obtained through the CASTEP program based on DFT. The Perdew–Burke–Ernzerhof (PBE) generalized gradient approximation (GGA) was selected as the exchange–correlation functional. The interaction between inner electrons and valence electrons was described by the OTFG ultra-soft pseudopotential.

The valence electronic configuration in the system are Sn 5s^2^5p^2^, O 2s^2^2p^4^, N 2s^2^2p^3^, C 2s^2^2p^2^, B 2s^2^2p^1^, and F 2s^2^2p^6^, S 3s^2^3p^4^. The calculation parameters of the doping system were as follows: The plane waves cut-off energy is 571.4 eV, the Brillouin zone *k*-space was divided by 2 × 3 × 8, the convergence accuracy of each atom was 10^−5^ eV/atom, the internal stress between atoms was less than 0.05 GPa, and the atom displacement was less than 0.0001 nm.

## Results and Discussion

### Structural optimization

Table 1 shows the results of geometric optimization of the crystal structure of SnO_2_ doped with non-metal elements. After replacing one O atom in SnO_2_, the crystal structure is distorted and the volume expands. The atomic radius of S is much larger than that of the other doping atoms resulting in a volume expansion of 8.315%. The lattice expansion rate also decreases in the sequence B > C > N. However, the F-doped SnO_2_ has a larger volume expansion. Therefore, it is necessary to analyze the bond formation before and after F atom doping, as shown in Table 2. The population value is usually used to present the bonding characteristics of crystals. In general, a strong covalent bond shows a high population value, while a strong ionic bond is characterized by a low value. The results in Table 2 reveal that after doping with heteroatoms, the population value of the Sn–O bond decreased, indicating that it to be more ionic. The population value of the Sn–F chemical bond is the lowest with only 0.125, showing strong iconicity, obvious electron localization, and a high electron affinity. The chemical bonds of other elements (C, B, N) and Sn elements exhibit the characteristics of covalent bonds. Therefore, SnO_2_ doped with F element exhibits an abnormal lattice expansion rate.

**Table 1:** Geometric optimization results of SnO_2_ with O substituted by a non-metal element.

	pure SnO_2_	SnO_2_/B	SnO_2_/C	SnO_2_/N	SnO_2_/F	SnO_2_/S

*a* (Å)	9.47454	9.74866	9.68215	9.66020	9.70223	9.74767
*b* (Å)	3.18638	3.24372	3.24956	3.24402	3.25942	3.25763
*c* (Å)	14.2118	14.6574	14.5507	14.4901	14.5451	14.6390
*V* (Å^3^)	429.048	463.456	457.802	454.088	459.934	464.725
Δ*V* (%)	—	8.02	6.702	5.836	7.1987	8.315

**Table 2:** Bond length and charge of the crystal structure with a non-metal atom replacing an O atom.

	average bond length (Å)				average net charge (*e*)		

	Sn–O	population value	Sn–M	population value	Sn	O	M

SnO_2_	2.054	0.505	—	—	2.07	−1.04	—
SnO_2_/F	2.096	0.472	2.289	0.125	1.9	−0.967	−0.58
SnO_2_/S	2.099	0.499	2.427	0.705	1.907	−0.966	−0.67
SnO_2_/C	2.095	0.5	2.186	0.885	1.918	−0.967	−0.75
SnO_2_/B	2.099	0.484	2.324	0.905	1.895	−0.964	−0.56
SnO_2_/N	2.091	0.496	2.105	0.695	1.964	−0.967	−0.95

Therefore, there are surplus electrons in F-doped SnO_2_ crystals. The average net charge of SnO_2_ before and after doping is also presented in Table 2. Except for N, which has a similar charge as O, other doping elements all have a lower charge. Especially, for fluorine and boron, the charge values were only half of that of oxygen. This indicates again that the doping of fluorine and boron elements can provide more surplus electrons to the system.

To evaluate the stability of the crystal structure of the doped lattice, it is necessary to calculate the defect binding energy of the lattice. It can be calculated according to Equation 1 [13]:

[1]
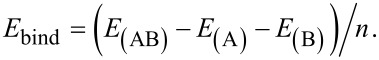


*E*_(AB)_ is the total energy of the doped structure, *E*_(A)_ and *E*_(B)_ are the chemical potentials of the atoms, and *n* is the total number of atoms in the unit cell structure. The total energy and binding energy of the doped structure are shown in Table 3.

**Table 3:** Binding energy of SnO_2_ with an O atom substituted by a non-metal element.

	total energy (eV)	concentration (atom %)	binding energy (eV)

SnO_2_/F	−3.55 × 10^4^	4.17	−5.38
SnO_2_/S	−3.52 × 10^4^	4.17	−5.24
SnO_2_/C	−3.50 × 10^4^	4.17	−5.27
SnO_2_/B	−3.49 × 10^4^	4.17	−5.22
SnO_2_/N	−3.51 × 10^4^	4.17	−5.33

The defect binding energy values of the doped systems (Table 3) are all negative, illustrating that all the doped crystal structures are stable structures. The defect binding energy decreases in the order of B, S, C, N, and F. The SnO_2_ doped with F has the lowest binding energy, which makes it the most stable structure.

### Band structure and density of states

**Figure 2:**
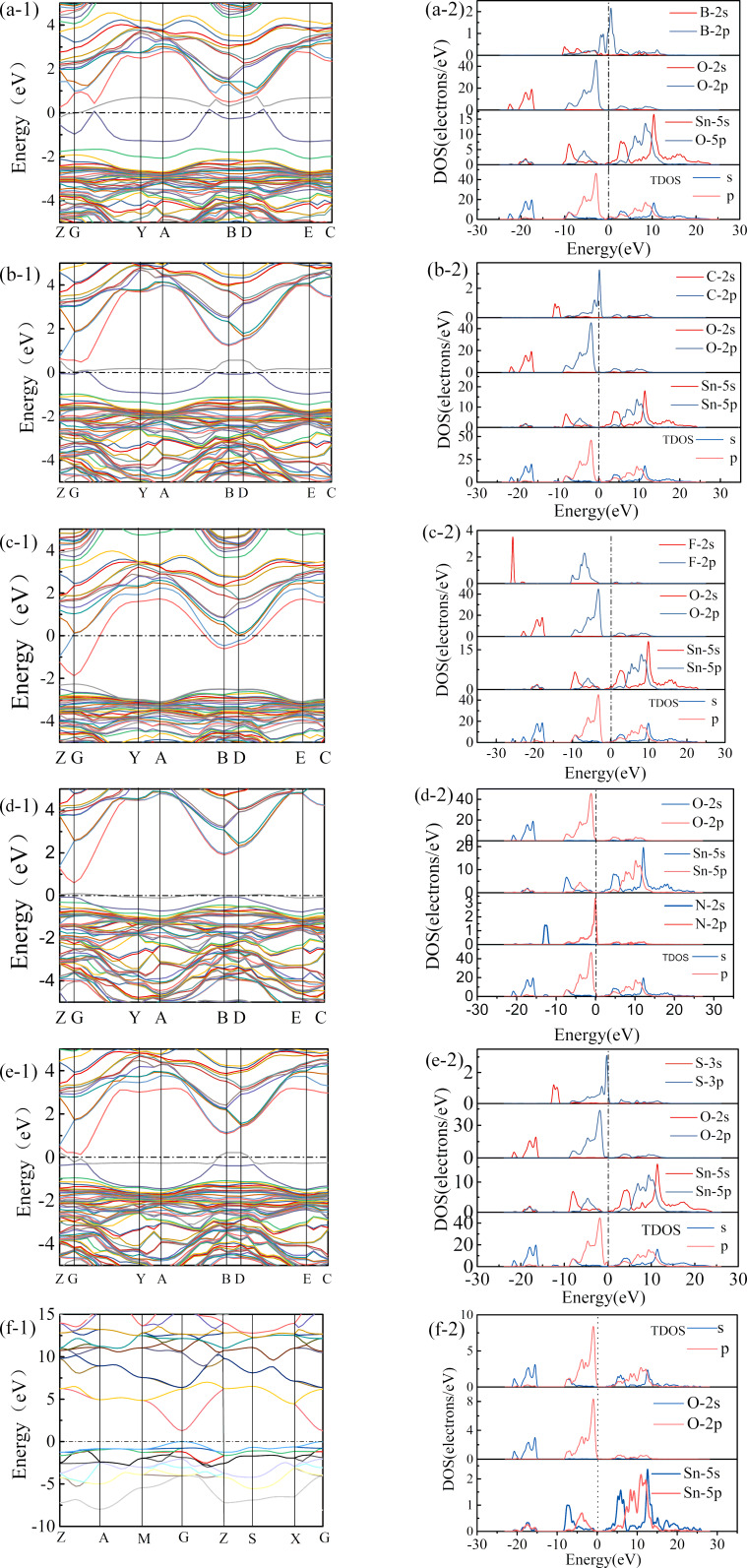
Energy band structure, total density of states and partial density of states of SnO_2_ doped with non-metal atoms: (a) SnO_2_/B, (b) SnO_2_/C, (c) SnO_2_/F, (d) SnO_2_/N, (e) SnO_2_/S, (f) SnO_2_.

After doping not only the crystal structure is distorted, but also the electronic structure of the SnO_2_ crystal is changed. The doping atoms introduce impurity levels in the bandgap of SnO_2_. The SnO_2_ crystal shows metallicity when the introduction of non-metal atoms causes the Fermi level to enter the conduction band. The electronic structure including the energy band structure, total density of states and partial wave state density of the doped system are shown in Figure 2.

For SnO_2_, the Fermi energy level is at the top of the valence band, indicating that the conductivity of SnO_2_ is low. The conduction band minimum (CBM) and the valence band maximum (VBM) are located at the same G point, which is consistent with the calculation results [14–15] indicating that SnO_2_ is a direct bandgap semiconductor. In this work, the calculated bandgap is 1.28 eV, which is consistent with previous calculation results [16–18]. However, the bandgap is lower than the experimental value of 3.6 eV [19], which is caused by the underestimation of the cross-correlation energy by the GGA function.

The energy of Sn 5s orbits and the interaction between Sn 5s and O 2p orbitals are overestimated, resulting in a wider valence band and narrower bandgap. The changes of bandgap and energy band cannot affect the electronic structure analysis of SnO_2_ crystals. The bandgap value can be modified by the complex variable function method (DFT + U) [20] to obtain a more accurate bandgap value. Although there are still some issues needed to be solved with this method, it is sufficient to mainly discuss the photoelectric properties of doped SnO_2_.

The total density of states of the SnO_2_ crystal shown in Figure 2 illustrates the that valence band of the system is divided into two parts, one from −19.1 to −14.9 eV and one between −8.1 and 0 eV. According to the density of partial wave states, the contributions to the deep energy level are from Sn 5s and O 2s, orbitals while the shallow energy level mainly consists of O 2p and Sn 5s orbitals with partial contribution of Sn 5p orbitals. The shallow energy level (from −8.1 to −5.8 eV) is mainly due to Sn 5s orbitals, and the O 2p orbital is responsible for the part between −5.8 and 0 eV. The main contributions to the conduction band are from Sn 5s and Sn 5p orbitals.

It is worth noting that the energy band of SnO_2_ changes significantly after doping. B 2p, C 2p, S 3p, and N 2p orbitals appear in the SnO_2_ bandgap. B, F and S cause the Fermi level of the doped crystal to move up. B and S introduce impurity levels in the SnO_2_ bandgap, which enhances the conductivity of the SnO_2_ crystal. The doping with B leads to more impurity levels in the forbidden band of SnO_2_, and the bandgap changes clearly, indicating that doping B atoms can adjust the SnO_2_ bandgap value well.

It can be seen from the partial wave state density diagram that the B 2p orbital and the S 3p orbital enter the SnO_2_ crystal bandgap. After the introduction of F atoms, the Fermi energy level passes through the conduction band of SnO_2_ crystal, and SnO_2_ becomes a conductor. The energy band structure of SnO_2_ doped with C and N shows that the Fermi level crosses the impurity level and the conductivity of SnO_2_ semiconductor is enhanced. To sum up, it can be seen that doping with F can enhance the conductivity of SnO_2_ crystals effectively.

**Figure 3:**
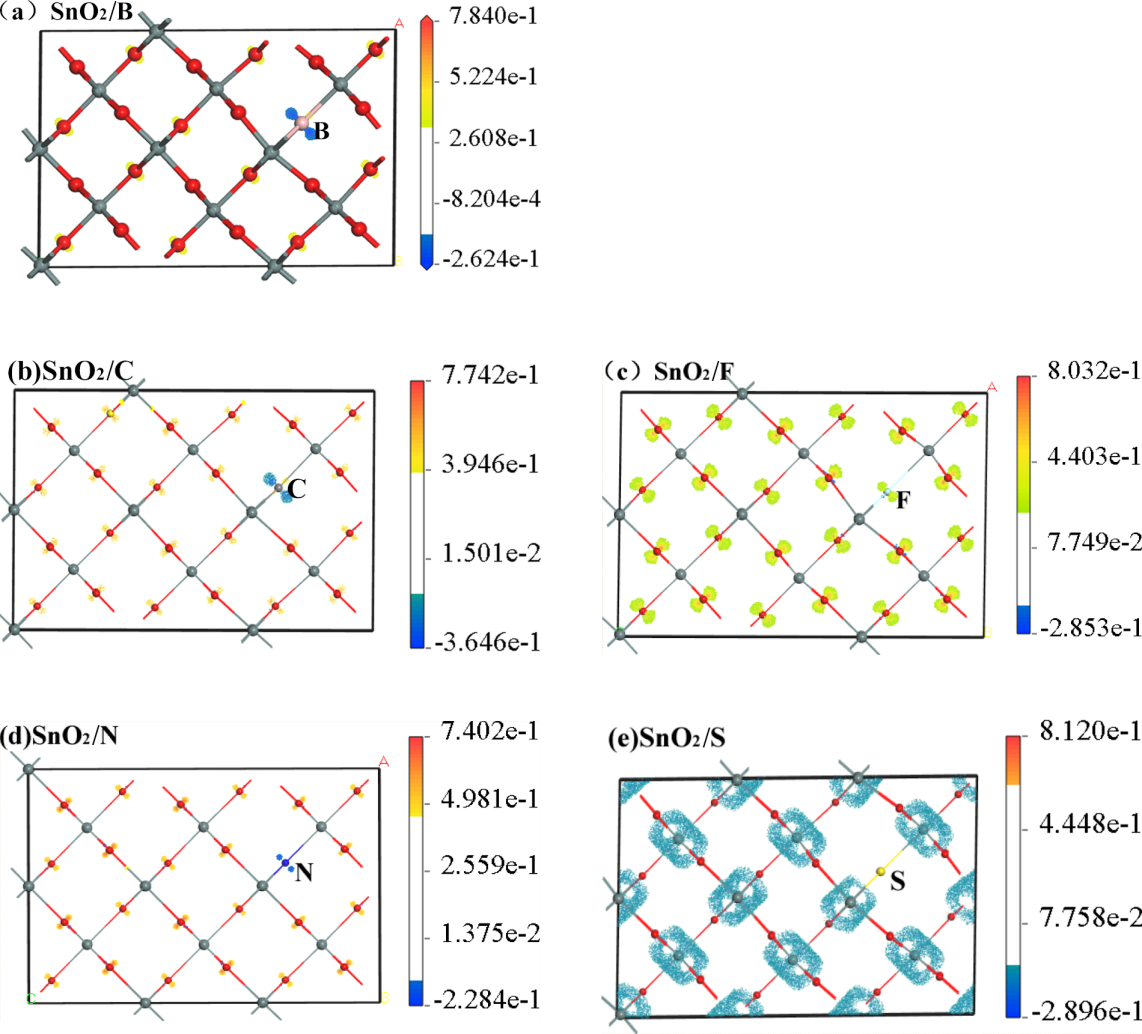
Differential charge density of SnO_2_ with O atom substituted by non-metal element.

In order to obtain information about charge transfer after doping, the secondary differential charge distribution of the elements was calculated and the results are shown in Figure 3. Compared with O, the ability of the doping atoms to accumulate charge is reduced. This provides more electrons as free carriers, making the system appear metallic. The analysis result is consistent with the analysis result of the energy band structure.

### Optical properties

SnO_2_ is widely used as a transparent conductive film material, especially in doors and windows, it is also preferred for coatings of low-emissivity glass. The most important property of low-emissivity is the reflectivity in the infrared region. According to the reflectance spectrum of the material, a good adiabatic behavior of the material requires the plasma frequency to be close to the visible region. Plasma is a system in which the dielectric constant is ε_r_ = 0, the concentration of positive and negative charges is the same, and positive and negative charges are free to move. When the probability of collision of free electrons in a solid is 0, the dielectric function is


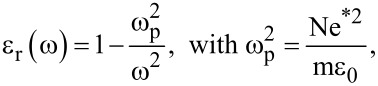


where ω_p_ is the plasma frequency. ω_p_ is the intrinsic characteristic frequency of the material. The plasma oscillation frequencies vary for different crystal materials. In general, it is proportional to the concentration of free electrons. When the vibration frequency of the incident light is greater than the plasma oscillation frequency, the crystal material is transparent. Otherwise, it is impossible for the light to pass through, showing metallic reflectivity.

**Figure 4:**
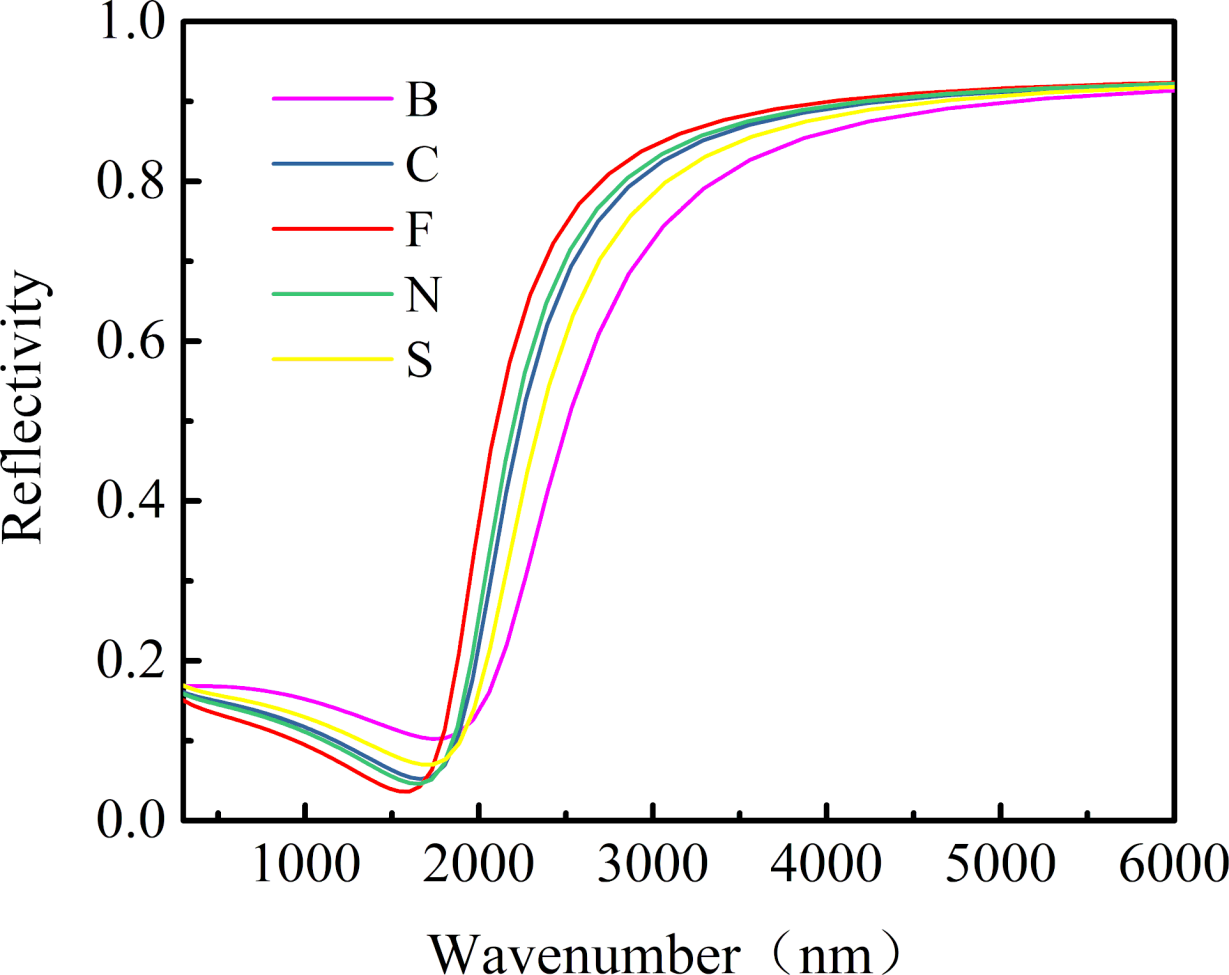
The reflection spectrum of SnO_2_ of O atom replaced by non-metal atom.

The reflectance spectra of SnO_2_ crystals doped with non-metal elements are shown in Figure 4. It shows that SnO_2_ doped with F had the highest reflectance, followed by doping with N, C, S, and B in this order. When the wavelength of light is greater than 1800 nm, the material shows metallic reflectivity, when the wavelength is below 1800 nm, the material shows transparency. Therefore, F-doped SnO_2_ is the most suitable material for low-emissivity glass coatings.

## Conclusion

Based on first-principles density functional theory, crystal structure, electronic structure, and optical properties of SnO_2_ doped with non-metal elements were theoretically analyzed. The calculation results confirm that the doping caused the crystal lattice to expand. The F-doped SnO_2_ lattice has the lowest binding energy and is prone to replacement doping. S-doping forms p-type semiconductors, and F-doping forms n-type semiconductors. The optical analysis results revealed that F-doped SnO_2_ possesses the highest reflectivity in the infrared region, and is most suitable as a low-emissivity coating material.
